# Evaluating Heat Stress in Occupational Setting with No Established Safety Standards Using Collective Data from Wearable Biosensors

**DOI:** 10.3390/s25061832

**Published:** 2025-03-15

**Authors:** Kyosuke Kato, Takuto Nishi, Sinyoung Lee, Li Li, Naoko Evans, Ken Kiyono

**Affiliations:** Graduate School of Engineering Science, Osaka University, Osaka 560-8531, Japan

**Keywords:** wearable sensor network, heart rate, heat stroke risk, Internet of Things, global warming

## Abstract

In recent years, living and occupational environments have been increasingly exposed to extreme heat. While the risk of heatstroke rises with greater heat stress, conventional knowledge and safety standards may no longer adequately assess heat stress under such extreme conditions. To address this issue, we propose a method for evaluating heat stress using collective data from wearable biosensors that monitor heart rate and physical activity in a group of workers. The novelty of this approach lies in utilizing collective data from wearable biosensors to assess environmental heat stress rather than individual health status. To quantify heat stress in specific environments or conditions, we introduce the heart rate response intercept, defined as the heart rate at 1 MET when the heart rate response to physical activity is approximated linearly. Using this heat stress index, we examined the effects of ambient temperature, aging, and obesity on heat stress. Our findings indicate that heat stress among obese workers was significantly high and should not be overlooked. Furthermore, because this method can quantify the effectiveness of heatstroke countermeasures, it serves as a valuable tool for improving occupational environments.

## 1. Introduction

As the global average temperature continues to break records, our living and occupational environments are increasingly exposed to unprecedented extreme heat [1,2]. The Sixth Assessment Report (AR6) of the Intergovernmental Panel on Climate Change (IPCC), published by the United Nations, estimates that the global average temperature rose by 1.09 °C between 2011 and 2020 compared to pre-industrial levels [3]. The report also indicates that temperatures over land are expected to rise 1.4 to 1.7 times faster than those near sea level. Additionally, the Japan Meteorological Agency (JMA)’s 2024 report on global temperatures estimates that the annual anomaly of the global average surface temperature for 2024 will be +0.62 °C above the 1991–2020 average. This would make 2024 likely the warmest year on record. The report also concludes that the past decade (2015–2024) is expected to be the warmest decade in the 134-year period since 1891 [4]. According to IPCC AR6, if greenhouse gas emissions continue to rise, global temperatures are projected to increase by 3.3 to 5.7 °C by the end of this century.

One of the most serious health risks associated with extreme heat is the increased morbidity and mortality from heat-related illnesses, such as heat exhaustion and heatstroke [5]. Heatstroke can be classified into two types based on its cause: (1) classic heatstroke and (2) exertional heatstroke [6]. Classic heatstroke results from prolonged exposure to high environmental temperatures combined with a reduced ability to dissipate heat. This type is more common among elderly individuals and patients with chronic diseases [7]. In contrast, exertional heatstroke is directly related to physical activity or work in a hot environment. Notably, it can occur even when ambient temperatures are not particularly high. This is often attributed to individuals pushing themselves beyond their physiological limits to meet external expectations. The primary mechanism of heatstroke involves a situation where heat gain exceeds heat loss, impairing the body’s ability to regulate temperature. This leads to an increase in core body temperature, triggering inflammatory responses and cytotoxic effects, which can ultimately result in multiple organ failure [8,9]. Severe cases of heatstroke require intensive care. However, it is important to emphasize that heatstroke can be prevented by implementing appropriate measures [7].

In areas where the maximum daytime temperature never exceeded 35 °C in the past, temperatures have recently exceeded human body temperature and even been exceeding 40 °C [10]. According to the United Nations, approximately 2.4 billion workers are currently exposed to excessive heat [11]. In general, increases in heat stress indices, such as maximum temperature and humidity, are strongly correlated with the risk of heatstroke, including its probability and severity [12]. For example, a 1 °C rise in temperature can increase mortality risk by 1% to 3% [13]. In occupational environments, several studies have demonstrated that excessive heat significantly elevates the risk of heatstroke, particularly among construction workers [14]. Despite dangerously hot occupational environments, most workers have no choice but to continue working there. To mitigate the risk of heatstroke, it is imperative that governments and companies take proactive measures to improve working conditions in high heat stress environments. Furthermore, researchers must propose effective, evidence-based strategies for heatstroke prevention.

To develop effective measures for heatstroke prevention, it is essential to properly assess heat stress and thermal strain associated with heatstroke. Rectal temperature, as a measure for core body temperature, is typically regulated to maintain a constant level of 37 °C, ensuring the stable functioning of vital organs such as the heart and brain [15]. It is well established that when a body’s heat balance breaks down and the core body temperature rises to 40 °C or higher, severe heatstroke can occur [16]. Past research has provided substantial findings on the relationship between rectal temperature and thermoregulatory functions, such as the ability to perspire [17]. Consequently, rectal temperature is widely regarded as a highly reliable indicator for assessing thermal strain. As a safety standard for rectal temperature in occupational environments, the American Conference of Governmental Industrial Hygienists (ACGIH) has established limits of 38 °C for workers who are not acclimatized to heat and 38.5 °C for those who are acclimatized [18]. These thresholds indicate when workers should stop working to prevent excessive thermal strain. Nonetheless, it is impractical for all workers to measure their rectal temperature during work and verify that it remains below the safety standard.

In actual occupational environments, rectal temperature measurement is rarely practical. Instead, heat stress is commonly assessed using the Wet-Bulb Globe Temperature (WBGT), as outlined in ISO 7243:2017 [19]. The WBGT incorporates factors such as air temperature, humidity, wind speed, and solar radiation, all of which influence heat stress. However, when using WBGT to assess heat stress, it is necessary to take into account variables such as the thermal resistance of clothing and workload, making it difficult to accurately assess heat stress on an individual basis. The WBGT-based heat stress safety standard is designed to maintain rectal temperature below 38 °C during continuous work. Nevertheless, the standard is largely based on Lind’s experiments from 1963, which have limitations. These experiments did not consider extremely hot environments exceeding human body temperature, nor did they include women or the elderly, as the study involved only two male participants [20,21]. Thus, it is important to recognize that there is insufficient evidence to support the applicability of the WBGT standard in cases of extreme heat that exceeds body temperature or among populations such as women, the elderly, children, or obese individuals. Moving forward, our challenge is to establish a heat stress assessment method that can reliably evaluate conditions in unprecedented extreme heat environments, which are beyond the scope of conventional standards.

Recent advances in IoT technology and wearable biosensors have enabled the collection of human biosignal data, such as heart rate and physical activity, without interfering with daily life or work activities [22]. Additionally, integrating these technologies with smartphones and cloud computing systems—known as cyber–physical systems—allows for real-time data collection and analysis [23]. Commercially available smartwatches, such as Fitbit and the Apple Watch, have already demonstrated the ability to measure heart rate and estimate physical activity [24,25]. These functionalities could potentially be applied to assess thermal strain [26]. Heart rate increases in response to heat stress, and this increase is strongly correlated with an increase in rectal temperature. ISO 9886:2004 provides standards for evaluating thermal strain on workers using heart rate measurement [27]. According to this document, the increase in heart rate induced by thermal strain is, on average, 33 bpm per 1 °C of increased body core temperature. Based on this, it is proposed that the upper limit for the increase in heart rate could be set at 33 bpm as a safety standard for workers.

It is important to distinguish between the concepts of heat stress and thermal strain [28]. Thermal strain refers to the physiological responses of the body to heat stress. The intensity of heat stress is quantified by measures such as WBGT, air temperature, wind speed, and relative humidity [29]. For individuals exposed to the same experimental conditions, the level of heat stress is identical. However, physiological responses, such as heart rate, rectal temperature, and sweating rate, are indicators of thermal strain and can vary from person to person, even under the same environmental conditions [30,31,32,33].

In this study, we propose a method to evaluate heat stress (as opposed to individual thermal strain) using the collective data of individuals equipped with wearable sensors capable of measuring heart rate and physical activity. While an increase in heart rate due to heat stress for an individual reflects thermal strain, an increase in the average heart rate of a group of workers in the same environment can be used as an indicator of heat stress specific to that environment. This is because the average heart rate reflects environmental characteristics rather than individual physiological variations. An increase in the mean heart rate of a group is strongly correlated with an increase in the mean rectal temperature of the group. Specifically, a heart rate increase of approximately 20 to 30 bpm corresponds to a 1 °C increase in rectal temperature [34,35,36,37]. By leveraging this relationship, we can assess the risk level of heatstroke by converting the observed increase in mean heart rate into a corresponding increase in rectal temperature, providing a reliable group-level evaluation of heat stress.

According to ISO 9886:2004, the individual heart rate can be modeled as

(1) $$\begin{array}{c} \mathrm{HR}={\mathrm{HR}}_{0}+\Delta {\mathrm{HR}}_{M}+\Delta {\mathrm{HR}}_{s}+\Delta {\mathrm{HR}}_{T}+\Delta {\mathrm{HR}}_{N}+\Delta {\mathrm{HR}}_{\varepsilon }, \end{array}$$

where ${\mathrm{HR}}_{0}$ is the mean heart rate of the subject at rest, $\Delta {\mathrm{HR}}_{M}$ is the increase in heart rate caused by work metabolism, $\Delta {\mathrm{HR}}_{s}$ is the increase in heart rate caused by static exertion, $\Delta {\mathrm{HR}}_{T}$ is the increase in heart rate associated with the thermal strain, $\Delta {\mathrm{HR}}_{N}$ is the increase in heart rate due to psychological factors, and $\Delta {\mathrm{HR}}_{\varepsilon }$ is the residual component in heart rate. For a group of workers, the individual variations in the components of Equation (1) are expected to approach zero or a constant value statistically when taking the group average. Therefore, the group-average heart rate can be approximated as

(2) $$\begin{array}{cc} E_{G}\left[\mathrm{HR}(M,H)\right] & =E_{G}\left[{\mathrm{HR}}_{0}\right]+E_{G}\left[\Delta {\mathrm{HR}}_{M}\left(M\right)\right]+E_{G}\left[\Delta {\mathrm{HR}}_{T}\left(H\right)\right]\\ \; & =f\left(M\right)+E_{G}\left[\Delta {\mathrm{HR}}_{T}\left(H\right)\right] \end{array}$$

where *M* and *H* represent the work metabolism and heat stress level, respectively; $E_{G}$ represents the operation taking the mean in a collective dataset of workers; and the function f(M) describes the heart rate depending on the work metabolism *M* without any heat stress.

**Figure 1:**
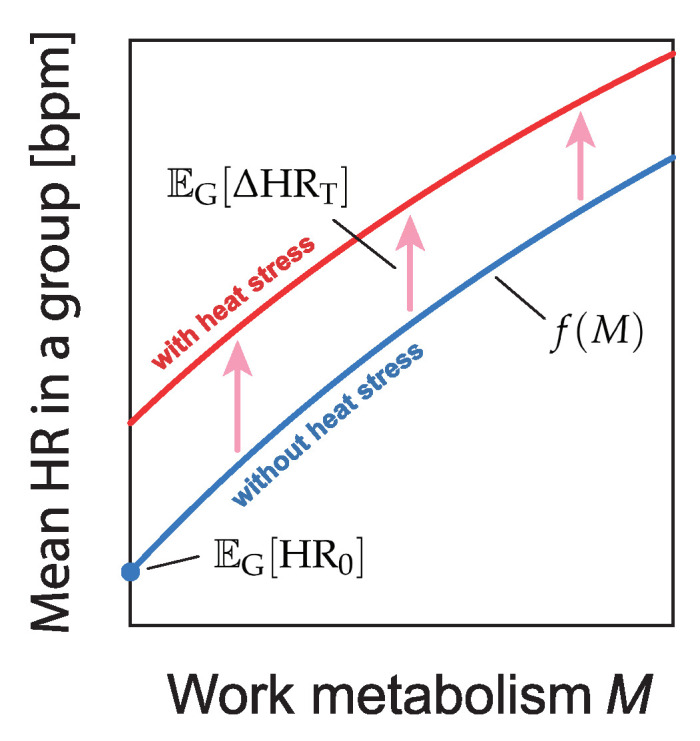
Schematic illustration of the group mean heart rate model described by Equation (2). Our approach quantifies thermal stress by estimating the shift in the plot between conditions without heat stress and those with heat stress.

**Figure 2:**
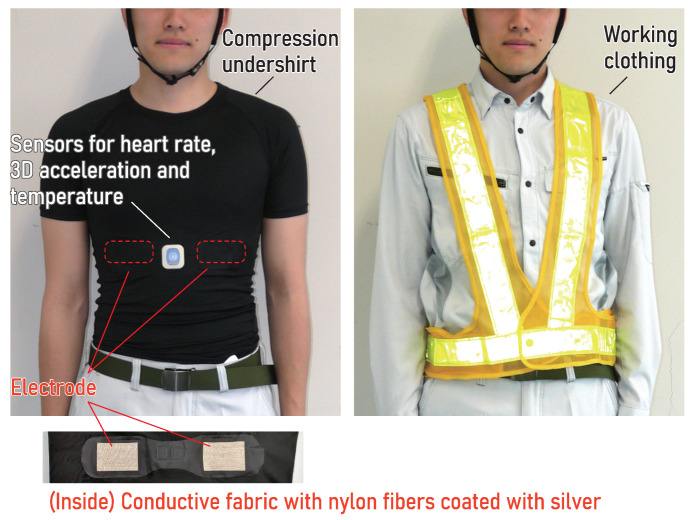
Undershirt-type heart rate monitor used for measurement (**left**) and typical work clothing for construction sites (**right**).

We propose a method to quantify heat stress by estimating $E_{G}\left[\Delta {\mathrm{HR}}_{T}\right]$ in Equation (2) (Figure 1) using real-world data collected via smart clothing (heart rate monitor in the form of an undershirt as shown in Figure 2). By analyzing data from 834 workers at actual construction sites, we quantified heat stress in relation to aging and body mass, factors that have not been accounted for in conventional heat stress assessments. This novel approach allows for a more comprehensive understanding of heat stress in occupational settings.

## 2. Materials and Methods

### 2.1. Subjects and Data

In this study, we reanalyzed the data collected during two summer periods from July to September 2018 and 2019. These data were stored in a cyber–physical system designed to integrate and analyze measurements from undershirt-type heart rate monitors (Figure 2), marketed under the name Smartfit for Work (www.smartfit.jp (accessed on 7 January 2025)). Smartfit for Work is a heat stress monitoring system for manual labor sites, developed by Kurabo, and is provided as a service to companies for real-time assessment of heat stress risks. In a clinical study on occupational diseases supported by the Japanese Ministry of Health, Labor, and Welfare, the system’s validity was verified, and it was evaluated as an effective tool for risk assessment in high-temperature working environments [38].

In this system, biosignal data, such as heart rate, were transmitted from the measurement device embedded in the undershirt to a smartphone carried by each worker. The smartphone processed the data, calculated minute-by-minute averages, and transmitted the results to a cloud-based system for further analysis.

The analyzed data were recorded throughout the full working hours of full-time employees. The dataset included heart rate, physical activity levels, and the temperature inside clothing, which were processed by the system every minute. Additionally, self-reported demographic data, including worker age, height, and weight, were used in the analysis. Heart rate was represented as the median value per minute, while physical activity was expressed as the mean per minute of the metabolic equivalent (MET) estimated using a tri-axis accelerometer (the estimation algorithm has not been disclosed). METs quantify the relative increase in oxygen consumption during physical activity compared to rest. Defined as oxygen intake per unit time per kilogram of body weight, 1 MET corresponds to 3.5 mL/kg/min at rest. Higher MET values indicate greater energy expenditure, with 2 METs representing twice and 3 METs three times the resting oxygen intake. Previous studies have demonstrated a strong correlation between high-frequency components of accelerometer data and METs [39,40]. Analyzing these components using a wearable accelerometer allows for reliable estimation of energy expenditure during activities such as walking and running, providing a practical approach for physical activity monitoring in research and clinical applications. Similarly, the temperature inside clothing was represented as the mean value per minute. The temperature sensor, positioned near the front surface of the chest-mounted device, does not directly contact the skin; instead, it measures the temperature inside the clothing, not the skin temperature.

In this study, we analyzed data from male workers aged 20 to 60 years who were employed in factories, construction sites, or the transport industry. Workers missing height or weight data, as well as those with a BMI below 10 kg/m^2^ or above 50 kg/m^2^, were excluded due to likely errors in the recorded height or weight values. Consequently, a total of 834 subjects were included in the analysis. We examined daytime data for each worker over a maximum period of three months during the summer. The characteristics of the subjects are summarized in Table 1 and Table 2.

This study was approved by the Ethics Committee of the Graduate School of Engineering Science at Osaka University. Consent to reuse the measured data for research purposes was obtained from all workers.

### 2.2. Data Analysis

To characterize the heart rate responses of the workers, we defined two indices: the heart rate response intercept ${\mathrm{HR}}_{1}$ and the heart rate response slope $\alpha_{\mathrm{HR}}$. These indices were derived by fitting a linear regression model to the relationship between heart rate and estimated workload determined by the accelerometer.

**Figure 3:**
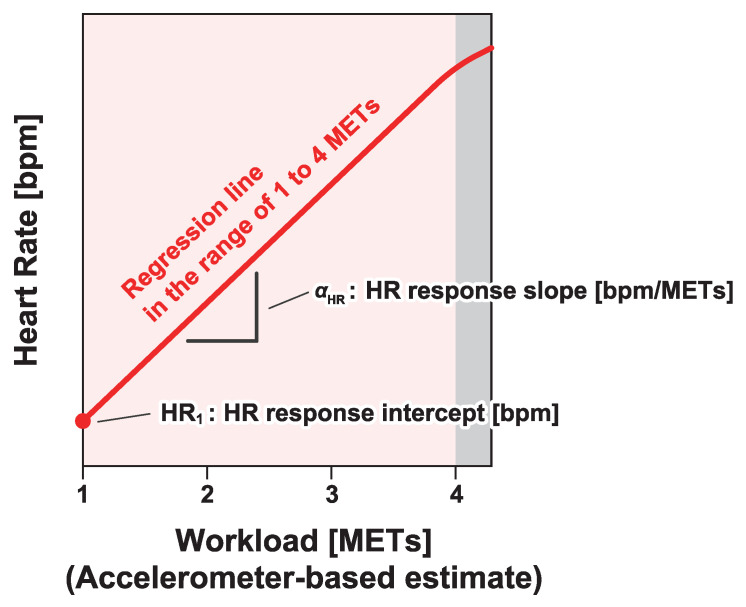
Definition of the heart rate response intercept and heart rate response slope. The heart rate response intercept ${\mathrm{HR}}_{1}$ and slope $\alpha_{\mathrm{HR}}$ are defined as the heart rate at 1 MET and as the slope, respectively, when the heart rate response to the physical activity is linearly approximated.

In the regression analysis, the median heart rate was calculated for each subinterval of the workload, with intervals set at every 0.25 METs. Then, a line was fitted to the median heart rate values plotted against workload [METs] in the range of 1 to 4 METs. Since most tasks during an 8-hour work shift remain below 4 METs to prevent excessive physical strain and fatigue, we limited our analysis to this range. Tasks at the 4 MET level correspond to approximately 30% of VO_2_ max, which is considered the upper limit for sustainable effort over a full workday [41]. In our data, the majority of work activities fell below this threshold, justifying our decision to restrict the regression analysis to values below 4 METs. As illustrated in Figure 3, the heart rate response intercept ${\mathrm{HR}}_{1}$ [bpm] was defined as the heart rate value on the regression line corresponding to 1 MET. Similarly, the slope of the line was defined as the heart rate response slope $\alpha_{\mathrm{HR}}$ [bpm/MET].

For intergroup comparisons, we applied the Dunn test with the Bonferroni correction to account for multiple comparisons.

## 3. Results

To begin, we describe the weather conditions that provide the context for the data we analyzed. In 2018, Japan experienced a significant number of days with extreme heat. For example, at the JMA observation site in Tokyo, there were 12 days when the maximum temperature exceeded 35 °C, while in Osaka, this number reached 27 days. The highest recorded temperature at the Tokyo observation site was 39.0 °C in July and 37.3 °C in August. Similarly, Osaka recorded a maximum temperature of 38.0 °C in July and 37.6 °C in August. According to a report by the Fire and Disaster Management Agency (FDMA) of the Ministry of Internal Affairs and Communications, the total number of people taken to hospital by ambulance due to heatstroke in Japan that year reached 95,137. This figure marked the highest number ever recorded at the time, though it was surpassed in 2024 when heatstroke-related emergency transports increased to 97,578.

In 2019, the frequency of days with maximum daytime temperatures exceeding 35 °C decreased slightly. Tokyo recorded 12 such days, while Osaka recorded 19. The highest temperatures at the Tokyo observation site were 34.6 °C in July and 35.6 °C in August. In Osaka, the maximum temperature reached 35.4 °C in July and 37.6 °C in August. Our data were obtained at various sites across Japan, including major cities such as Tokyo and Osaka.

**Figure 4:**
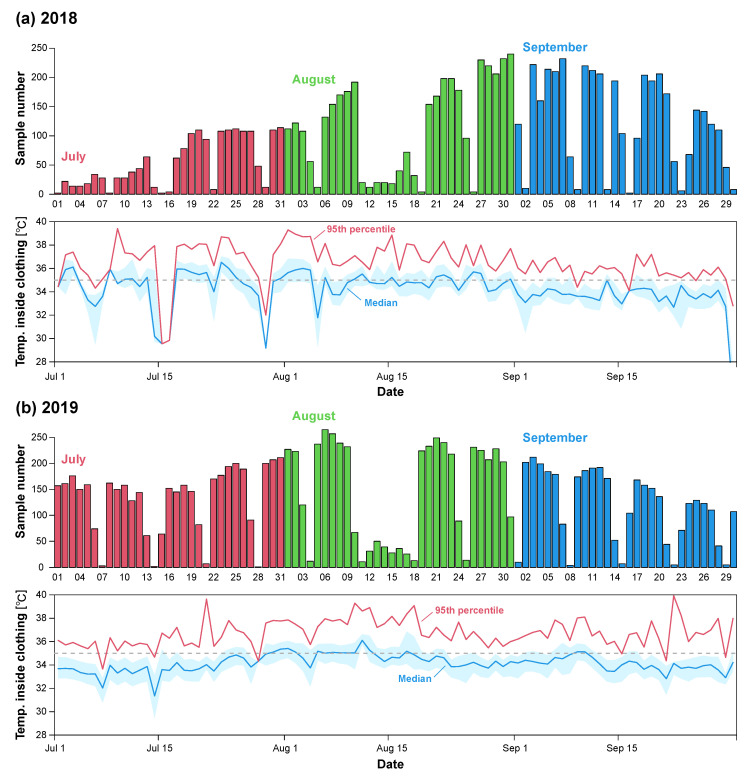
Daily sample numbers (**upper panel**) and temperatures inside the clothing (**lower panel**) for 2018 (**a**) and 2019 (**b**). The dataset for these two years comprises observations totaling 21,166 days. The temperature inside the clothing was calculated by identifying the maximum daily temperature for each worker. The median (solid blue line), interquartile range (shaded light blue area), and 95th percentile (solid red line) of these daily maximum temperatures were then calculated across all workers. The dashed lines in each lower panel of (**a**,**b**) indicate 35 °C.

The daily number of samples (i.e., the number of workers for whom measurement data were available) and a summary of the temperatures inside the clothing (the maximum daily temperature for each worker) are shown in Figure 4. On days with a large sample size, data from more than 200 workers were included, as shown in the upper panels of Figure 4a,b. In 2018, Japan experienced a record-breaking heatwave, with many days in July and August when the temperature inside the clothing exceeded 35 °C for more than half of the workers, as shown in the lower panel of Figure 4a. In the following sections, we analyze all of the data collected.

### 3.1. Effect of Temperature Inside Clothing on Heat Stress

To illustrate the potential application of our approach, we estimated the dependence of the heart rate response intercept ${\mathrm{HR}}_{1}$ and slope $\alpha_{\mathrm{HR}}$ on the temperature inside clothing. Our analysis revealed that, in some cases, the temperature inside the clothing exceeded 37 °C. This finding suggests that the ambient temperature in real-world occupational settings can surpass body temperature. While no large-scale laboratory experiments have been conducted under such extreme heat conditions, our data demonstrate that workers are exposed to these extreme environments in real-world occupational contexts.

**Figure 5:**
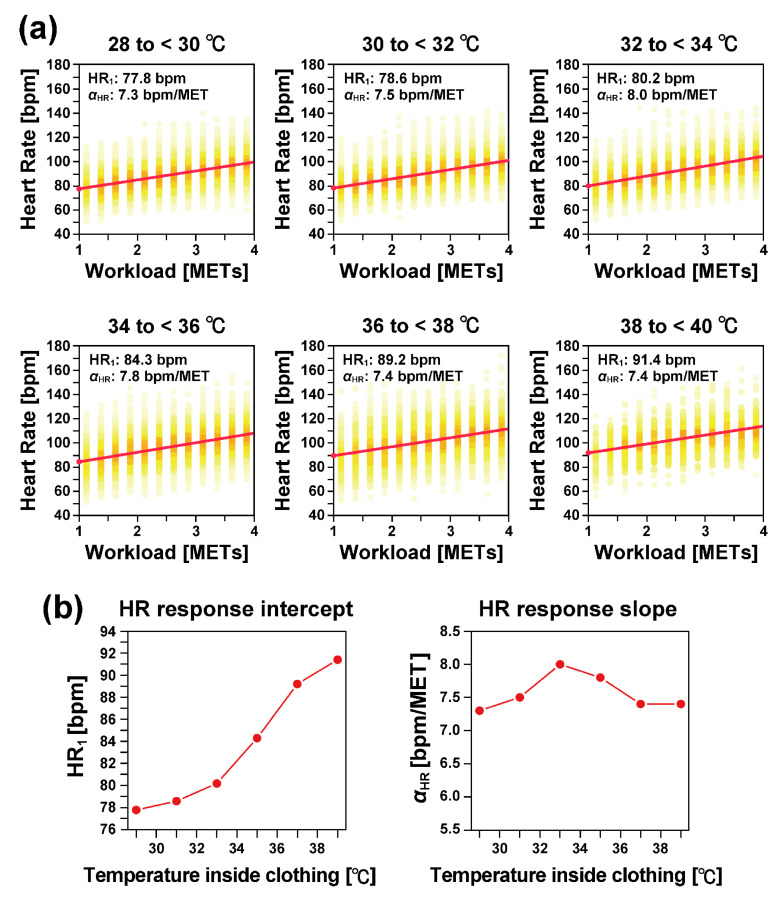
(**a**) Heart rate response depending on temperature inside clothing. The data were divided into 2 °C intervals between 28 °C and 40 °C based on the temperature inside clothing. The colors represent the density of data points in each subinterval of the workload, where orange indicates high density, and light yellow indicates low density. The regression lines are shown as solid red lines. (**b**) The left panel illustrates the temperature dependence of the heart rate response intercept ${\mathrm{HR}}_{1}$, and the right panel shows the dependence of the slope $\alpha_{\mathrm{HR}}$.

We divided the data based on the temperature inside clothing into six temperature categories: (1) 28 °C or higher but less than 30 °C (28 to <30 °C), (2) 30 °C or higher but less than 32 °C (30 to <32 °C), (3) 32 °C or higher but less than 34 °C (32 to <34 °C), (4) 34 °C or higher but less than 36 °C (34 to <36 °C), (5) 36 °C or higher but less than 38 °C (36 to <38 °C), and (6) 38 °C or higher but less than 40 °C (38 to <40 °C). Additionally, we estimated ${\mathrm{HR}}_{1}$ and $\alpha_{\mathrm{HR}}$ in each temperature category (Figure 5). As shown in Figure 5a, the heart rate response to workload can be well approximated by a straight line in all temperature categories.

As illustrated in Figure 5b, the heart rate response intercept (${\mathrm{HR}}_{1}$) increased monotonically as the temperature inside the clothing increased. Specifically, ${\mathrm{HR}}_{1}$ increased by 13.6 bpm when the temperature increased by 10 °C, from 29 °C to 39 °C.

In contrast, the heart rate response slope ($\alpha_{\mathrm{HR}}$) showed almost no dependence on the temperature.

### 3.2. Effect of Aging on Heat Stress

Knowledge about the effects of aging on heat stress and strain in previous studies has been limited and insufficiently quantitative. To address this gap, we analyzed the data by dividing the participants into age groups. Specifically, we categorized the data into four age groups: (1) 20 to 29 years (20 s), (2) 30 to 39 years (30 s), (3) 40 to 49 years (40 s), and (4) 50 to 59 years (50 s).

**Figure 6:**
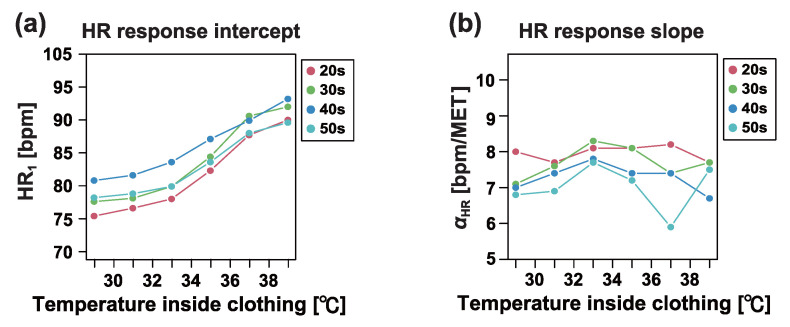
(**a**) Dependence of heart rate (HR) response intercept ${\mathrm{HR}}_{1}$ on temperature inside clothing in each age group. (**b**) Dependence of heart rate (HR) response slope $\alpha_{\mathrm{HR}}$ on temperature inside clothing in each age group. See Table 1 for the age group definitions and number of workers in each age group.

Figure 6 illustrates the changes in the mean values of heart rate response indices across age groups, depending on the temperature inside the clothing. Our results did not reveal any monotonic or systematic trends associated with age. Although ${\mathrm{HR}}_{1}$ differed across age categories, there was minimal age dependence on the temperature-induced increase in ${\mathrm{HR}}_{1}$.

**Figure 7:**
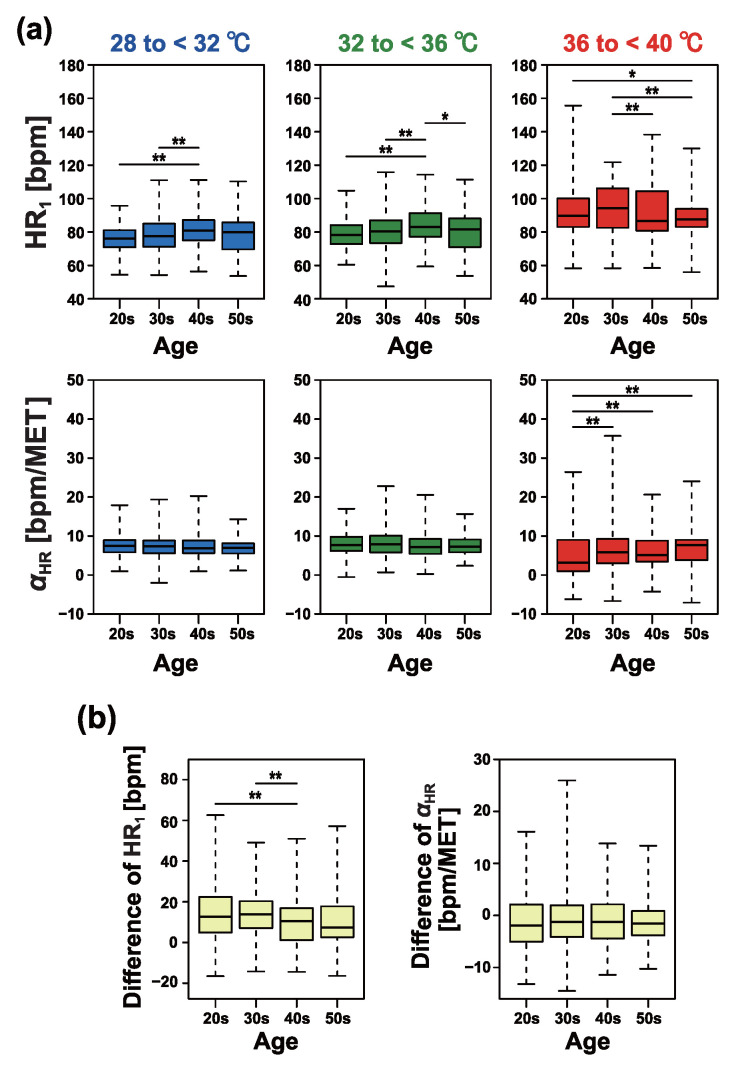
Comparison of heart rate response intercepts and slopes estimated from individuals. (**a**) Results of comparing age groups for each temperature condition. (**b**) Differences in heart rate response intercepts (**left**) and slopes (**right**) between conditions for 28 to <32 °C and for 36 to <40 °C. The asterisk indicates the *p*-value according to the Dunn test with Bonferroni correction (* *p* < 0.05, ** *p* < 0.01).

Figure 7 presents detailed comparisons of the individual differences between age groups. While some comparisons produced small Bonferroni-corrected *p*-values, no consistent monotonic or systematic trends based on age were observed.

As shown in Figure 7b, we further analyzed the differences in the indices ${\mathrm{HR}}_{1}$ and $\alpha_{\mathrm{HR}}$ caused by increases in clothing temperature. Specifically, we calculated the differences in each index between two temperature conditions: 28 to <32 °C and 36 to <40 °C. The relative changes in both indices did not indicate a need to consider age-related differences among workers aged 20 to 59 years.

### 3.3. Effect of Obesity on Heat Stress

It has been noted that obese individuals may be particularly vulnerable to hot environments [42,43]. However, the increase in heat stress associated with obesity has not been systematically quantified, and no standards have been established for estimating heat stress in obese workers. To address this issue, we investigated the effects of overweight and obesity on heat stress in real-world work environments. According to the current BMI classification standards [44], we categorized the data based on BMI into four groups: (1) less than 18.5 kg/m^2^ (underweight: UW); (2) 18.5 kg/m^2^ or higher but less than 25 kg/m^2^ (healthy weight: HW); (3) 25 kg/m^2^ or higher but less than 30 kg/m^2^ (overweight: OW); and (4) 30 kg/m^2^ or higher (obesity: OB).

**Figure 8:**
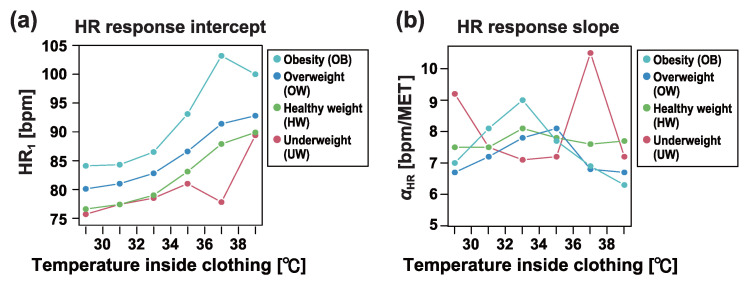
(**a**) Dependence of heart rate (HR) response intercept ${\mathrm{HR}}_{1}$ on temperature inside clothing in each BMI category. (**b**) Dependence of heart rate (HR) response slope $\alpha_{\mathrm{HR}}$ on temperature inside clothing in each BMI category. See Table 1 and Table 2 for the BMI category definitions and number of workers in each BMI category.

Figure 8 illustrates the changes in ${\mathrm{HR}}_{1}$ and $\alpha_{\mathrm{HR}}$ as a function of the temperature inside the clothing across different BMI categories. As BMI increased and the tendency toward obesity became more pronounced, ${\mathrm{HR}}_{1}$ also increased. Moreover, in the obesity category, the increase in ${\mathrm{HR}}_{1}$ associated with rising temperature was greater than in the other categories.

**Figure 9:**
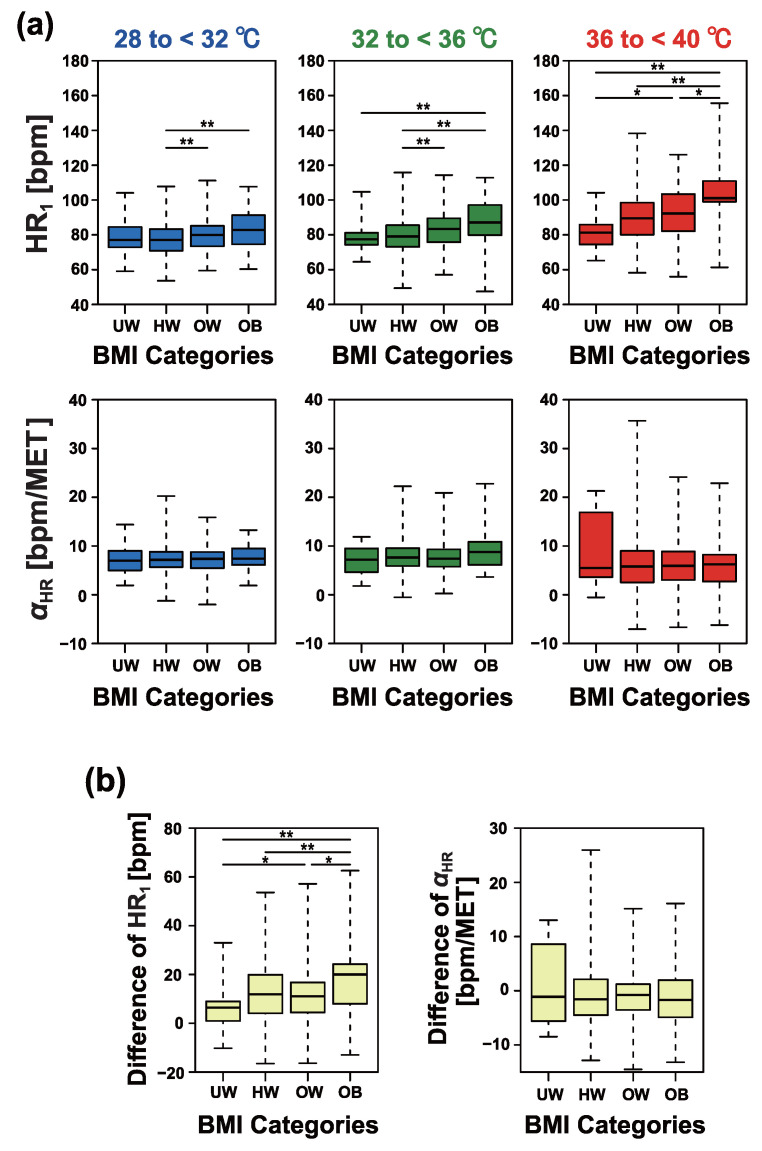
Comparison of heart rate response intercepts and slopes estimated from individuals. (**a**) Results of comparing BMI categories for each temperature condition. (**b**) Differences in heart rate response intercepts (**left**) and slopes (**right**) between conditions for 28 to <32 °C and for 36 to <40 °C. UW, HW, OW, and OB represent, respectively, underweight (less than 18.5 kg/m^2^), healthy weight (18.5 kg/m^2^ or higher and less than 25 kg/m^2^), overweight (25 kg/m^2^ or higher and less than 30 kg/m^2^), and obesity (30 kg/m^2^ or higher). The asterisk indicates the *p*-value according to the Dunn test with Bonferroni correction (* *p* < 0.05, ** *p* < 0.01).

Figure 9 provides detailed comparisons of individual differences between BMI categories. As shown in Figure 9a, under conditions of 32 to <36 °C and 36 to <40 °C, ${\mathrm{HR}}_{1}$ exhibited a monotonic increase with increasing BMI categories. In contrast, $\alpha_{\mathrm{HR}}$ showed no significant dependence on BMI.

As shown in Figure 9b, we further analyzed the differences in the indices ${\mathrm{HR}}_{1}$ and $\alpha_{\mathrm{HR}}$ induced by BMI. Specifically, we calculated the differences in each index between two temperature conditions: 28 to <32 °C and 36 to <40 °C. The relative changes in ${\mathrm{HR}}_{1}$ demonstrated a significant increase in obese workers.

## 4. Discussion

Our study demonstrated that the level of heat stress could be evaluated using the collective heart rate data of workers in real-world environments, even under conditions not fully addressed by conventional knowledge and safety standards. This approach represents a novel method for building real-world evidence in occupational settings, as opposed to traditional laboratory-based experimental methods.

Well-designed laboratory experiments offer advantages, such as facilitating statistical inference and providing controlled environments for interpreting observed results. Nonetheless, they also have notable limitations, including small sample sizes, discrepancies between experimental conditions and real-world environments, and difficulty addressing VUCA (Volatility, Uncertainty, Complexity, and Ambiguity) issues in a timely manner.

In contrast, our approach, which integrates and analyzes data from wearable biosensors distributed in work environments (a wearable biosensor network), has several advantages. It enables the assessment of actual working conditions, facilitates the collection of large-scale data, and allows for rapid risk assessments even in the face of sudden environmental changes. Importantly, this approach focuses on utilizing biosignal data collected from wearable biosensors, not for individual monitoring, but for safety assessments of groups working in the same environment. This provides a new perspective on the applications of wearable sensors in occupational settings.

However, real occupational environments are characterized by significant variability in workers’ personal characteristics and job types. This heterogeneity can result in considerable variation in the observed results, which potentially complicates interpretation. To address this challenge, improving the reliability of statistical estimates by increasing the sample size is essential. One potential strategy to achieve this is by analyzing previously accumulated data from the target environment.

While increasing the number of workers included in the analysis is essential to some extent for improving statistical reliability, equipping every worker with a wearable sensor is often neither practical nor cost-effective, particularly in large-scale occupational settings such as construction sites, factories, or outdoor event venues. Instead, collecting and analyzing data from a fixed or randomly selected subset of workers can provide an effective means of quantifying heat stress in the occupational environment. This targeted data collection strategy significantly reduces costs compared to equipping all workers with wearable sensors, while still offering reliable insights into environmental heat stress.

This study evaluated the effects of ambient temperature (temperature inside clothing), aging, and obesity on heat stress. In our approach, the increase in heat stress was quantified by the increase in ${\mathrm{HR}}_{1}$, estimated as the average heart rate response of a group of workers. The level of risk was then assessed by converting this increase in ${\mathrm{HR}}_{1}$ into an approximate increase in rectal temperature. According to ISO 9886:2004, an increase in heart rate of 33 bpm is associated with a 1 °C rise in core body temperature, although no supporting references were provided. In a separate analysis of 9046 ICU patients, it was reported that a 1 °C increase in core temperature corresponded to an increase in heart rate of 9.46 bpm in women and 7.24 bpm in men [45]. These relatively small increases in heart rate may be attributed to heat production that is not associated with muscle activity. Conversely, studies based on heat stress experiments involving exercise have shown that a 1 °C increase in rectal temperature corresponds to an increase in heart rate of 20 to 30 bpm [34,35,36,37].

Although there is no scientific consensus on the quantitative relationship between increases in heart rate and rectal temperature, for the purposes of safety assessment, we adopt a conservative estimate: an increase of 20 bpm in heart rate corresponds to an approximate increase of 1 °C in rectal temperature. Using this interpretation, our findings indicate that the resting heart rate increases by 10 bpm when the temperature inside clothing rises from 30 °C or less to approximately 38 °C. This corresponds to an approximate increase in rectal temperature of 0.5 °C. For obese workers, under the same conditions, the resting heart rate increases by 20 bpm, which corresponds to an approximate increase of 1 °C in rectal temperature. If the safety standard is to limit the increase in rectal temperature to below 1 °C, these results suggest that additional safety measures are necessary for obese workers. Regarding the effect of aging, our analysis found no clear evidence to indicate that age needs to be considered as a significant factor for workers under 60 years of age.

It is important to note that our estimates reflect the effects of the heat stroke countermeasures implemented in each workplace. Many workplaces now adopt various measures to prevent heat stroke, such as extending the duration of breaks, increasing the frequency of breaks, ensuring breaks are taken in cool environments, and providing cold drinks during breaks. Conventional heat stress evaluations using WBGT only account for environmental factors and cannot capture the effects of these countermeasures. In contrast, our approach, which measures group heart rates, can quantify the impact of heat stroke countermeasures as a reduction in ${\mathrm{HR}}_{1}$. This enables us to evaluate not only heat stress levels but also the effectiveness of workplace interventions aimed at mitigating risks. Another advantage of our method is its ability to quantify the improvements in workplace safety and conditions, offering a practical tool for risk reduction and environmental enhancement in occupational settings.

Our study has several limitations. First, it only addressed temperature inside clothing as a climatological factor, making it impossible to examine the relationship with other environmental factors, such as humidity or wind speed. In recent years, electric-ventilated work clothes equipped with fans have become more common. Even when the temperature inside clothing is similar, the humidity and perceived wind speed may vary significantly. Future research should analyze both temperature and other environmental factors inside clothing to provide a more comprehensive evaluation of heat stress. In this study, we used a cyber–physical system for data measurement and storage. This system is also capable of integrating information from environmental IoT sensors. In future research, we aim to generate real-world evidence on the effects of heat stress by incorporating environmental parameters such as WBGT, air temperature, relative humidity, and wind speed.

Second, the accuracy of the MET estimation using a tri-axis accelerometer requires further validation, particularly for physically demanding tasks. Although acceleration-based MET estimation has been shown to be highly accurate for activities such as walking and running [39,40], it is not suitable for work involving tasks such as lifting or carrying heavy objects. Therefore, the MET estimation algorithm should be refined to account for different types of work and industry-specific demands.

Third, our study did not sufficiently incorporate individual characteristics. Although temperature is a critical factor in heat stress, previous studies have highlighted that work style and health conditions also play significant roles [46]. Unfortunately, we were unable to collect information on subjects’ health, such as medical history or hospital visits. A key challenge for future studies will be to assess heat stress while considering individual health conditions.

Finally, the workers in our study were male, leading to a lack of analysis on female workers. In recent years, women have increasingly participated in various sectors, including those involving work in hot environments. Given the growing number of women in such roles, it is important to expand the scope of research to include female subjects. There are well-documented gender differences in heart rate responses to increases in core body temperature, underscoring the need for further analysis focusing on female subjects.

All conventional standards for hot occupational environments have been established based on experiments conducted with male subjects. To ensure occupational safety for both sexes, it is essential to develop standards specifically for females, and our approach can contribute to this effort. In this study, we used undershirts designed for males, resulting in the absence of data for females. However, similar undershirt-type heart rate monitors have been developed for females, making it possible for future studies to evaluate female workers using these garments.

## 5. Conclusions

This study analyzed heart rate monitoring data collected in real-world occupational settings, including factories, construction sites, and the transportation industry. To quantify the level of heat stress in a given environment or condition, we proposed the heart rate response intercept ${\mathrm{HR}}_{1}$, defined as the heart rate at 1 MET when the heart rate response is approximated linearly. Using this metric, we evaluated the effects of ambient temperature (temperature inside clothing), aging, and obesity on heat stress. Our findings highlighted that heat stress among obese workers was significantly elevated and could not be ignored, underscoring the need for additional safety measures tailored to obese workers.

This approach enables the assessment of heat stress levels in real-world environments and conditions that are not fully addressed by conventional knowledge or safety standards. As such, it has practical applications for preventing heatstroke and improving workplace environments to enhance worker safety and well-being. 

## Figures and Tables

**Table 1:** Subjects’ characteristics.

	Overall
**Variables**	(***n*** = **834**)
Age (year), median (IQR)	36 (27–45)
Groups of age	
20 s (20 to 29 years)	277 (33.2)
30 s (30 to 39 years)	244 (29.3)
40 s (40 to 49 years)	205 (24.6)
50 s (50 to 59 years)	108 (12.9)
Male sex	822 (98.6)
Height (cm), median (IQR)	170 (167–175)
Weight (kg), median (IQR)	67 (60–75)
BMI (kg/m^2^), median (IQR)	23.0 (21.1–25.2)
BMI Categories	
Underweight (<18.5 kg/m^2^)	27 (3.2)
Healthy weight (18.5 to <25 kg/m^2^)	575 (68.9)
Overweight (25 to <30 kg/m^2^)	187 (22.4)
Obesity (≥30 kg/m^2^)	45 (5.4)

Data were presented as unweighted number (percentage) of subjects unless otherwise indicated. Abbreviation: IQR, interquartile range.

**Table 2:** BMI distribution by age group.

	BMI (kg/m^2^)			
Groups of Age	<18.5	18.5 to <25	25 to <30	≥30
20 s (20 to 29)	12 (4.3)	222 (80.1)	33 (11.9)	10 (3.6)
30 s (30 to 39)	6 (2.5)	159 (65.2)	69 (28.3)	10 (4.1)
40 s (40 to 49)	8 (3.9)	125 (61.0)	52 (25.4)	20 (9.8)
50 s (50 to 59)	1 (0.9)	69 (63.9)	33 (30.6)	5 (4.6)

Data are presented as unweighted number (percentage) of subjects unless otherwise indicated.

## Data Availability

Data are unavailable due to privacy or ethical restrictions.

## Abbreviations

The following abbreviations are used in this manuscript:

IPCC	Intergovernmental Panel on Climate Change
JMA	Japan Meteorological Agency
WBGT	Wet-Bulb Globe Temperature
UW	underweight
HW	healthy weight
OW	overweight
OB	obesity
